# Oral health related quality of life (OHRQoL) following third molar surgery in Sub-Saharan Africans: an observational study

**DOI:** 10.11604/pamj.2016.25.97.7656

**Published:** 2016-10-19

**Authors:** Ramat Oyebunmi Braimah, Kizito Chioma Ndukwe, Foluso John Owotade, Stephen Babatunde Aregbesola

**Affiliations:** 1 Department of Dental and Maxillofacial Surgery, Usmanu Danfodio University Teaching Hospital, Sokoto, Nigeria; 2 Department of Oral & Maxillofacial Surgery and Oral Pathology, Obafemi Awolowo University Teaching Hospitals Complex, Ile-Ife Osun State, Nigeria

**Keywords:** Eating, oral health, physical, psychological, quality of life, social, symptom, surgicalextraction, third molar surgery

## Abstract

**Introduction:**

Surgical extraction of the impacted third molar is one of the commonest minor oral surgical procedures carried out in oral surgery. Problems created by the disturbances in post-extraction wound healing and physiologic sequelae of third molar surgery can significantly affect the patient's quality of life.

**Methods:**

The study population consisted of 135 subjects that required surgical extraction of mandibular third molar under local anesthesia and met the inclusion criteria. Patients were assessed pre-operatively and post-operatively on days 1,3,5,7, and 14 using the United Kingdom Oral Health related Quality of Life questionnaire (UK-OHRQoL).

**Results:**

This study also showed that surgical removal of impacted teeth exerted a negative influence on patient's Quality of life (QoL) across various physical, social, psychological aspects of life. UK-OHRQoL-16 mean scores showed that severe difficulty in eating was experienced by 106 (78%) patients on postoperative day (POD) 1. The symptom however improved within the first week with only 16 (11.9%) experiencing this symptom by POD 7 and none by POD 14.

**Conclusion:**

There was a deterioration in oral health related quality of life in the immediate postoperative period particularly POD 1 and 3 following third molar surgery, which slowly returned to preoperative level by 7th day. Routines such as eating, laughing and smiling, work and speech were also affected. Patients need to be informed of these symptoms after third molar removal so as to enable them prepare very well for the procedure and its sequelae.

## Introduction

Quality of life (QoL) is a term widely used in oncology to assess social well-being and the effects of treatment on cancer patients [1]. It has also become widely applied to other fields in health care including dental practice [1]. The term QoL was originally indexed in the Index Medicus in 1975, however over 5000 articles have been published with the concept of QoL [1]. In dental practice, QoL as it relates to oral health has only been recently employed in dental care [2]. Presently, QoL assessment is now regarded as an important component for assessing dental care outcomes [3]. Extraction of the impacted third molar is one of the commonest minor oral surgical procedures carried out in oral surgery [4]. The most common postoperative complications following third molar removal are alveolar osteitis and surgical site infection [5, 6]. Others are permanent nerve damage [7, 8], pain, trismus, swelling, and difficulty in swallowing [9]. Although a minor surgical procedure, the global cumulative cost of extraction of third molar runs into billions of dollars [4]. The impact of oral diseases and oral procedures on QoL is very obvious following third molar surgery. Similarly problems created by the disturbances in post-extraction wound healing and physiologic sequelae of third molar surgery can significantly affect the patient's QoL [10, 11]. Before consenting to surgery, patients are informed of the risks and benefits of having their third molars removed. Most of the information available to both clinicians and patients focuses on clinical outcomes [12]. Although this information is important, patients want to know about the surgical procedure and expectations during recovery. Increasingly, there have been calls for greater understanding of the effects of third molar removal on patients' day-to-day living, as there are few such studies published [13]. In Sub- Saharan Africa literature search has revealed no such study. The aim of this study therefore was to evaluate the QoL among outpatients who have undergone surgical extraction of their lower third molars from an African point of view.

## Methods

A prospective study conducted in the Department of Oral and Maxillofacial Surgery, Obafemi Awolowo University Teaching Hospitals Complex (OAUTHC), Ile-Ife, Osun State between May 2011 and February 2012. A total of 135 healthy patients, 18-35 years old, including 63 males and 72 females volunteered with a written document to partake in the trial. The criterion for including a patient in the study was individual healthy subject that presented in the Oral and Maxillofacial Surgery clinic for the surgical extraction of an impacted lower third molar tooth under local anesthesia and willingness to give a written informed consent. Exclusion criteria include Subjects with history suggestive of underlying systemic diseases e.g. diabetes mellitus, congestive cardiac failures, chronic nephritis, chronic liver disease, systemic malignancy, sickle cell disease, presence of acute pericoronal infection, subjects that are immune-compromised ,subjects that require antibiotic prophylaxis for endocarditis, subjects with history of allergy to Penicillins, subjects with dyspeptic symptoms or who are being treated for peptic or duodenal ulcer disease, pregnant subjects and breastfeeding mothers. After consenting to participate in the study and before removal of indicated third molars, subjects baseline (demographics) comprising of age, gender, ethnicity, marital status, education, indication for seeking third molar removal and income level. QoL was assessed pre-operatively using the 16 item United Kingdom Oral Health Related Quality of Life measure (UK-OHRQoL) [14].

All surgery was performed by the same surgeon using a standardised procedure. Under local anaesthesia (2% lignocane with 1:100,000 adrenaline), a buccal three-sided mucoperiosteal flap was raised by a gingival margin incision around the mandibular second and third molars with anterior and posterior relieving incisions using a #15 surgical blade. Bone removal was done by bucco-distal guttering technique using fast hand piece (80,000-150,000 rev/mins) and #10 surgical round cutting bur under continuous irrigation with sterile 0.9% saline solution. Tooth sectioning when indicated was performed with a tapering fissure bur in a fast hand piece (80,000-150,000 rev/mins) under irrigation with sterile 0.9% saline solution. After tooth removal by the use of Coupland elevators, the alveolus was inspected, curetted for granulation tissue removal (for those with associated periapical granuloma and cysts) and irrigated with sterile saline solution. In addition, the flap base was carefully debrided and irrigated with sterile normal saline solution. A 3/0 black braided silk suture material was used to close the wound without tension.

All patients were placed on the same analgesic tabs ibuprofen 400mg 8hourly for 3 days and Amoxyl/Clauvulanic acid 625mg 12hourly for 5days. A review appointment was scheduled for postoperative days 1, 3, 5, 7 and 14. On each of these days subjects were asked to complete the questionnaire (UK-OHRQoL) [14]. For UK-OHQoL-16, there are 4 domains: 1) symptom level; 2) body function level; 3) at person level; 4) at social level.

These domains have several symptoms that were assessed as shown in Appendix 1. Each item was scored: very bad effect- score 1; bad effect- score 2; no effect- score 3; good effect- score 4; very good effect- score 5.

Total scores range from 16 to 80. 16 was the worst possible score (very bad effect across all 16 aspects of life quality) and 80 indicated the best possible oral health (very good effect across all 16 aspects of life quality).

### Statistical analysis

Data analysis was carried out using Stata 10 (Statacorp College Station, Texas). Descriptive statistics was carried out for socio-demographic variables such as age, gender, marital status and occupation. The descriptive variables that are continuous parameters such as mean, median, minimum, maximum and measures of variability were determined. For descriptive variables that are categorical, simple frequency and percentages were determined. Statistical analysis was done using intention- to- treat analysis [15]. The psychometric properties of the United Kingdom Oral Health Related Quality of Life instrument were evaluated by means of internal reliability (Cronbachs' α).

## Results

Cronbach's α were calculated for all the domains of the UK-OHRQoL instrument, and high values were obtained. For the symptom level domain it was 0.89, for the body function level domain it was 0.81, for the personality level domain it was 0.82, and for the social function level domain it was 0.81. A total of 135 subject participated and no patient was excluded from the study. Ninety one completed the study at post operative day 14. The number of patients assessed at each study visit is shown in Table 1. Among the study participants 53.3% were females with mean age of 24.4±5.4 and 46.7% were males with mean age 25.3±4.4 (Table 2). The preoperative clinical status is shown in Table 3. In the immediate postoperative period after surgical extraction of the mandibular third molar there was a decrease in the QoL measure as compared to the preoperative status. The mean values for UK-OHRQoL-16 scores are presented in Table 4.

**Table 1:** Number of patients accessed at each visit

	Number of patient
Pre-op	135
Post-op Day 1	135
Post-op Day 3	132
Post-op Day 5	132
Post-op Day 7	132
Post-op Day 14	91

**Table 2:** Sex distribution by mean age of patients

	Freq (%)	Age range	Mean ±SD
Female	72 (53.3)	18-35	24.4±5.4
Male	63 (46.7)	18-35	25.3±4.4
Total	135 (100)		

**Table 3:** Pre-operative clinical status of patients

Pre-operative Clinical Status	Freq (%)
**Indication for extraction**	
Pericoronitis	100 (74.1)
Apical periodontitis	35 (25.9)
**Total**	**135 (100)**
**Impaction type**	
Mesioangular	70 (51.9)
Distoangular	29 (21.4)
Vertical	19 (14.1)
Horizontal	17 (12.6)
**Total**	**135 (100)**

**Table 4:** Distribution of mean quality of life score in domains at pre-operative and post-operative days

	Pre-op	Post-op Day 1	Post-op Day 3	Post-op Day 5	Post-op Day 7	Post-op Day 14
Symptom level	5.36±0.97	5.18±0.90	5.72±0.78	5.95±0.7	6.02±0.45	6.13±0.45
Body function level	13.98±1.63	11.98±1.78	13.42±1.91	14.69±1.16	14.91±1.24	15.22±1.03
Personality level	14.22±1.60	13.91±1.56	14.68±1.32	15.05±0.81	15.07±1.09	15.48±3.24
Social level	11.41±1.27	11.06±1.21	11.48±1.35	11.88±1.00	12.06±0.88	12.09±0.59

UK-OHRQoL-16 (Table 5) mean scores showed that severe difficulty in eating was experienced by 106 (78%) patients on postoperative day (POD) 1. The symptom however improved within the first week with only 16 (11.9%) experiencing this symptom by POD 7 and none by POD 14. Speech was affected in 75 (55.6%) of the patient on POD 1 and by POD 7 only 4 (2.9%) complained about this symptom. Bad breath was experienced in 23 (17.0%). Seventy-two (53.3%) patients could neither laugh nor smile properly on POD 1, this however improved as only 7 (5.2%) experience this symptom by POD 7. Sleep was affected in 51 (37.8%) of patients on POD 1 this however reduced to only 1 patient complaining of this symptom by POD 7. Fifty (37.0%) patients could not go to work on POD 1 and 35 (25.9%) of the patient could not function very well socially. Disturbance in romantic relationship was reported in 17 (12.6%) of the patients on POD 1 with the number decreasing to only 1(0.7%) by POD 5 and none by POD 7.

**Table 5:** Oral health related quality of life (OHRQoL) -16 scale

OHRQoL-16 SCALE	
Symptom level	Comfort breath odour
Body function level	Eating
	Appearance
	General health
	Speech Smiling/laughing
At person level	Relax/sleep
	Confidence
	Mood
	Carefree manner Personality
At social level	Social life
	Romantic Relationship
	Work/usual jobs
	Finance
Key for scoring Symptoms	Score
Very bad	1
Bad	2
No effect	3
Good effect	4
Very good effect	5

## Discussion

Recovery for health-related quality of life (HRQoL) measures includes subjects' perception of recovery, which in turn includes return to a usual lifestyle and recovery of oral function [14]. Results from this study showed that the surgical removal of impacted teeth exerted a negative influence on patient's QoL across various physical, social, psychological aspects of life such as limitation in daily routine, ability to chew food, ability to open mouth, ability to speak, comfort, laughing and smiling and sleep. Similar to this study, Mc-Grath et al [16] showed deterioration in QoL in the immediate postoperative period following third molar surgery as measured by OHIP-14 scores and OHQoLUK-16. Colorado et al [1] also concluded that lower third molar surgery significantly influences patient's QoL, especially during the first 3 days of postoperative period. Also , White et al [17] reported that the median number of days required to return to daily activity and social life after third molar surgery was three days with recovery for chewing and return to regular diet taking five to seven days respectively [17]. Observations from this study are similar as subject recovery started by POD3 as evident by Mean QoL returning to preoperative period after third molar removal.

Inability to work after surgical removal of mandibular third molars has been reported by Berge TI in a study of 201 patients. In the study, 115 (57.2%) had their work compromised in the study on POD 1 [18]. Similar to our result where 50 (37.0%) reported inability to work, has shown that lost work due to mandibular third molar removal is enormous. Our study could not provide the financial implication of this lost work; however in the Berge's study the financial implication of lost work secondary to mandibular third molar removal has been estimated to be 46.4 million NOK per year local currency in Norway [18].

To the best of our knowledge no study on QoL after third molar surgery was found in Nigeria and Sub Saharan Africa. Despite this paucity of literature on QoL after third molar removal in Nigeria and sub Saharan Africa, this study observed that QoL was severely compromised especially in the first three postoperative days after mandibular third molar surgery. The limitation of this study is that the study is from only one centre in Nigeria, however, it could serve as baseline data from this part of the world. Further studies from other centers are needed to substantiate these results.

## Conclusion

There was a significant deterioration in OHRQoL in the immediate postoperative period particularly POD 1 and 3 following third molar surgery, which slowly returned to preoperative level by 7^th^ day. Patients undergoing mandibular third molar extraction should be adequately informed of the possible sequelae of the procedure on their quality of life especially in the immediate postoperative period to know what to expect and how to cope with such when they arise.

### What is known about this topic

- Third molar surgery is the commonest minor oral surgical procedures in Oral and Maxillofacial Surgery;
- Normal physiologic sequelae and complications of mandibular third molar removal affect patients' quality of life;
- Removal of lower third molar leads to loss of work hours which has been estimated to run into millions of dollars.

### What this study adds

- In Sub- Saharan Africa literature search has revealed no such study; hence this study will serve as baseline data in which other studies in African can be based on;
- The questionnaire used in the present study (UK-OHRQoL-16) was based on the new WHO model of health: *“structure-function-activity-participation”* focusing on both disease and health states, while previous studies on quality of life used questionnaires was based on a previous World Health Organization's (WHO) model of health: *disease-impairment-disability-handicap*, focusing on the burden of disease;
- This study also showed that surgical removal of mandibular third molar can affect sexual relationships.
